# A Novel Method for Obtaining Analytical Parameters Based on Double-Flank Measurement

**DOI:** 10.3390/s24092734

**Published:** 2024-04-25

**Authors:** Xiaoyi Wang, Mingkang Liu, Tianyang Yao, Kunlei Zheng, Chengxiang Zhao, Longyuan Xiao, Dongjie Zhu, Zhaoyao Shi

**Affiliations:** 1 School of Mechatronics Engineering, Henan University of Science and Technology, Luoyang 471003, China; kklmk163@163.com (M.L.); yty0101@139.com (T.Y.); zkl15538694420@163.com (K.Z.); zcx_98@126.com (C.Z.); xly15137615751@163.com (L.X.); zdjwsbn@163.com (D.Z.); 2 Henan Key Laboratory of Mechanical Design and Transmission System, Henan University of Science and Technology, Luoyang 471003, China; 3 College of Mechanical & Energy Engineering, Beijing University of Technology, Beijing 100124, China; shizhaoyao@bjut.edu.cn

**Keywords:** gear metrology, double-flank rolling tester, analytical parameters, double-flank measurement

## Abstract

Double-flank measurement is the most commonly used method for full inspection of mass-produced gears and has high measurement efficiency, but it cannot obtain the analytical parameters and is not helpful enough to evaluate the NVH performance of the gears. Based on the double-flank rolling tester with a new principle, a simulation method for double-flank measurement and a solving method for analytical parameters are proposed. Using the simulation method, the double-flank measurements without random error can be obtained through the collision detection algorithm. The solving method uses the iteration to obtain the minimum rolling length of each position of the tooth surface, then obtains the analytical parameters of the gear. In the experiments, the difference between the profile deviations obtained by the solving method and superimposed in the simulation method is less than 0.03 μm. The experiment results have verified the correctness of the simulation method and the solving method. These methods can greatly improve the value of double-flank measurement.

## 1. Introduction

The popularization and development of new energy vehicles pose new challenges to gear transmission systems, including high speed, high precision, and low noise requirements. As they are the most important part of gearbox, the design, manufacture, and measurement analysis of gears are crucial. Traditional measurement methods are very useful for evaluating gear machining accuracy, but cannot efficiently provide comprehensive measurement data for noise analysis of mass-produced gears [1]. 

To analyze and evaluate noise, it is necessary to obtain analytical parameters, which include parameters such as pitch deviations, profile deviations, and helix deviations. The measurement methods for gear analytical parameters are mainly divided into two categories. One is contact measurement methods based on the Gear Measuring Center (GMC) or Coordinate Measuring Machine (CMM), and the other is optical or visual non-contact measurement methods, such as point laser measurement, line laser measurement, laser holographic measurement, computer tomography (CT) measurement, etc. [2]. Reference [3] introduces the contact measurement technology and optical measurement technology in gear measurement, pointing out that gear measurement requires innovation in mathematical flank description, superficial probing, and evaluation methods.

The commonly used instruments for contact measurement methods are the GMC and CMM, as well as the Gear Integrated Error (GIE) measurement instrument.

The GMC is the integrated application of information technology, computer technology, and CNC technology for gear measurement instruments. It boosts the characteristics of high measurement accuracy, comprehensive functions, and strong universality. The GMC based on coordinate measurement is a milestone in the development of gear measurement instruments and is gradually becoming the main trend of gear measurement instrument development [4].

The CMM is also an important instrument for measuring analytical parameters. As a universal, highly automated, and highly accurate measurement system, the CMM has been widely used in industrial production and scientific research [5]. By contrast, traditional CMMs are limited by factors such as ball size and probe system performance, and they fail to meet the accuracy measurement requirements of analytical parameters for small-module gears. Therefore, Werth has launched a micro-optical fiber probe. With a ball diameter reaching a minimum of 20 micrometers [6], it expands the measurement range of the CMM for gear analytical parameters.

GIE measurement technology can also obtain analytical parameters. The GIE measurement instrument usually uses a teeth-skipped worm as the measuring element, which can quickly obtain various individual error information and comprehensive error information on the same instrument [4].

There is also a lot of research on non-contact measurement methods for gear analysis parameters. HEXAGON has developed an optical one-dimensional displacement sensor called HP-O. As a non-contact sensor, it can be applied to the CMM to achieve scanning measurement of flanks. The comparative experiment on the optical measurement effect of HP-O in reference [7] shows the potential of the new optical probe to improve scanning speed and point density. By utilizing this new optical probe, more information about the geometric deviation of the flanks can be obtained. 

Reference [8] proposes a three-dimensional point cloud measurement system based on a line-structured light sensor and a high-accuracy air floating rotary table. The measured three-dimensional point cloud data are used to calculate profile deviations and pitch deviations and then are compared with traditional contact measurements. The results show good consistency between the measured values of the structured light sensor and the reference measurement values. Another non-contact measurement method is gear visual inspection, which mainly has two purposes: accuracy detection and defect detection, which can be applied in detection scenarios with mass-produced and difficult clamping [2]. The visual inspection equipment in reference [9] can detect errors such as geometric dimensions, radial runout, and span measurement, as well as surface defects such as missing teeth and black spots, which can help to determine product quality. 

However, existing gear analysis measurement methods and instruments are usually very inefficient and cannot meet the needs of mass-produced gears measurement. Therefore, for the quality control of mass-produced gears, the main methods currently used are gear composite deviation measurements (including single-flank composite deviation measurement and radial composite deviation measurement). Among them, the double-flank test is the most commonly used inspection method due to its high efficiency. However, traditional double-flank measurement data analysis methods cannot be used to obtain analytical parameters (only radial composite deviations can be obtained) [10,11,12], making them ineffective for the analysis and evaluation of gear transmission NVH performance. At present, there is a large amount of relevant literature focusing on how to improve the measurement accuracy and efficiency [13,14,15,16,17,18,19], but few involve methods for how to obtain analytical parameters based on double-flank measurements.

With a single-tooth rack probe, references [20,21] successfully obtain analytical parameters in double-flank measurement, but this rack probe cannot achieve continuous rotation measurement like conventional double-flank measurement. Additionally, when measuring gears with a large number of teeth, the efficiency is low. In a word, currently, there is no relevant research on the method for obtaining analytical parameters based on the double-flank measurements of the master gear and the product gear [22].

Therefore, in order to study how to acquire analytical parameters based on double-flank measurement, obtaining double-flank measurements with high accuracy is of top priority. However, in the actual double-flank rolling tester measurement process, the measurements contain errors from various sources, including errors of the master gear and the axial runout of the double-flank rolling tester, which can cause obstacles to the analysis of the double-flank measurements. The double-flank measurement simulation method can avoid the interference of master gear errors, axial runout errors, and environmental errors in the test, and obtain more accurate double-flank measurements for the analysis in this paper.

There are several studies on simulation methods for double-flank measurement. In reference [23], a set of theoretical systems for real flank meshing was studied, which used the algorithm for finding the contact point through a tangent to solve the calculated and theoretical values of the contact point on the flank contact line. Aiming to create a digital simulation algorithm for double-flank measurement, the research team from Beijing University of Technology proposed to apply the principle of minimum directed distance to solving the double-flank instantaneous meshing point, and realized the simulation analysis of gear double-flank measurement to meet the requirements of simulation accuracy [24]. In reference [25], the meshing characteristics of the digital flank of spiral bevel gears were investigated through the minimum rotation angle method, and the authors conducted contact analysis of gear pairs with high-accuracy approximation of the actual flank. The profile deviations and pitch deviations were simulated and measured based on the rack probe in the literature [26]. However, there is no literature on a double-flank simulation method to analyze the gear pair of master gear and product gear with errors. 

The author’s team studied the measurability of analytical parameters and the influence factors of measurable area based on the double-flank measurement [22]. However, there is no effective method in the relevant literature for obtaining analytical parameters by using double-flank measurements of the master gear. In addition, the existing simulation methods for double-flank measurement are generally for the rack probe or the gears without error, and there is no effective double-flank test simulation method for the gear pair of master gear and product gear with errors. This paper puts forward a simulation method to obtain the measurements of the double-flank test of gears with errors and a method to obtain analytical parameters based on double-flank measurements and carries out simulation tests. In the experiments, analytical parameters are obtained by using double-flank measurements, which verifies the correctness of the methods proposed in this paper.

## 2. Simulation Method of Double-Flank Measurement

### 2.1. Method

**Figure 1:**
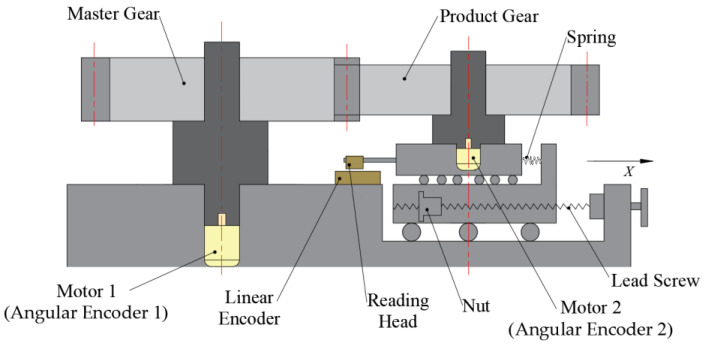
Schematic diagram of new principle of double-flank rolling tester.

The double-flank simulation method in this paper is proposed based on the principle of the measurement device in Figure 1. Figure 1 shows a double-flank rolling tester, in which the master gear and product gear make a double-flank meshing motion, that can obtain analytical parameters. The master gear is driven by motor 1 to make rotary motion. The master gear has only one rotational degree of freedom on the base, and its rotational angle information is measured by the angular encoder of motor 1. The product gear is coaxial with motor 2, the product gear has only one degree of freedom of rotation on the sliding platform, and its rotational angle information is measured by the angular encoder of motor 2. Motor 2 can also provide loading torque during the measurement process, facilitating the exploration of double-flank measurements under loading conditions. The screw–nut pair can adjust the position of the product gear in the *X*-axis direction. When the center distance between the master gear and the product gear in the double-flank movement is changed, the sliding platform can be translated in the *X*-axis direction, and the *X*-axis displacement information is measured by the linear encoder. The sliding platform is subjected to spring force, which keeps the gear pair in a double-flank meshing state during the gear meshing transmission process.

The simulation acquisition method for the double-flank measurements based on the above measurement device principle is as follows.

Knowing the parameters of the master gear and the product gear, as well as the profile deviations of the product gear, establish the tooth flank equations of the master gear and the product gear with errors (Equation (4)). Given the angle of rotation of the two gears and the center distance, make the two gears be in the mesh position under the same coordinate system by means of the coordinate transformation matrix (Equation (5)). Given a large initial center distance to make the two gears be in a non-contact state, reduce the center distance with a fixed step size and find the center distance position where the left and right tooth circumferential backlash of the two gears involved in meshing is zero.

The method of determining whether the circumferential backlash between the left and right tooth flanks of the two gears involved in meshing is zero is as follows: By orthogonal decomposition of the profile deviations, the coordinate set *P_n_* is obtained by stacking the profile deviations of each sampling point on the product gear flank. Then, obtain the set of normal equations *N_n_* (Equation (7)) of the sampling points on the flank of the product gear. The coordinate set of the sampling points on the master gear flank after coordinate transformation is *M_n_*. Fit the point set *M_n_* to obtain the fitting curve *L_Mn_*. Solve the intersection set *S_en_* of *N_n_* and fitting curve *L_Mn_*. Based on the positional relationship between the *S_en_* and the *P_n_*, determine the positional relationship between the master gear and the product gear with errors. According to this positional relationship, find the critical point where double-flank measurement occurs without interference. The corresponding sensor data when the gear pair is at the critical point position are recorded, and double meshing measurement data of one angle position are obtained.

After these steps, the double-flank measurements of one angle position of the product gear are obtained. Change the rotation angle of the product gear and repeat the above process so that double-flank measurement data of a sufficient number of angle positions can be obtained.

After the measurement of one of the measured flanks of the product gear is completed, rotate the two gears, change the measured flank, and repeat the above double-flank simulation process. Then, the double-flank measurements of all flanks of the product gear can be obtained.

**Figure 2:**
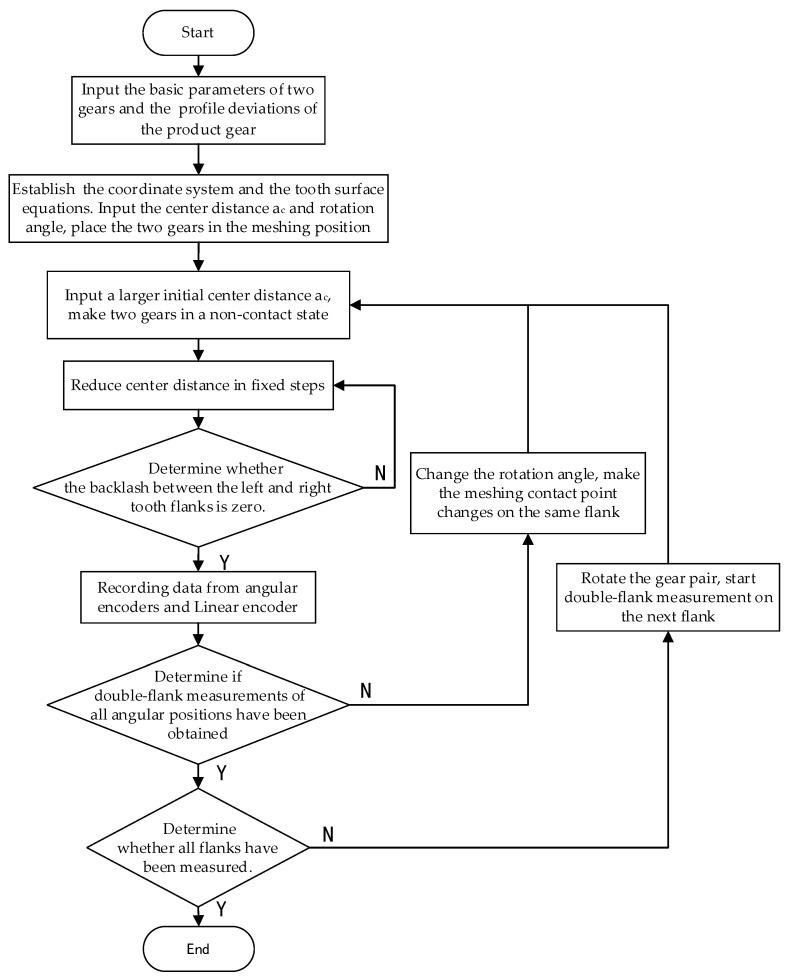
Flow chart of the simulation method for obtaining double-flank measurements.

The simulation calculation process is shown in Figure 2.

**Figure 3:**
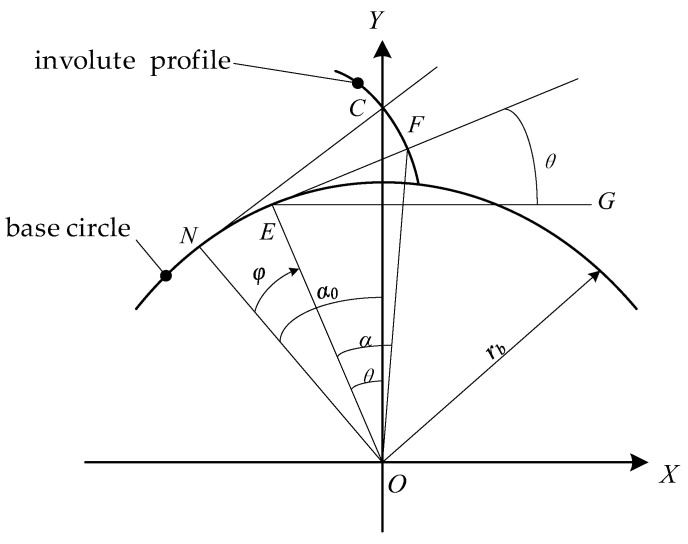
Gear coordinate system.

The coordinate system used to establish the tooth flank equations is shown in Figure 3. The coordinate system takes the center of the involute cylindrical gear as the origin and the gear axis as the *Z*-axis; the positive direction of the *Z*-axis is perpendicular to the outward direction of the paper surface; the direction of the ray passing through the pitch point from the origin is the positive *Y*-axis; and the *X*-axis is determined according to the right-hand rule. Two parameters (*Z*, *α*) determine any point on the flank, *Z* represents the coordinates in the Z direction, and *α* represents the transverse pressure angle at any given point on the flank. When the flank rotates around the *Z*-axis, *φ* represents the angle of rotation from the position shown in Figure 3. The *N* point in Figure 3 is the tangent point between the tooth profile’s normal and the base circle. The transverse pressure angle at pitch point *C* is $\alpha_{0}$. Any point on the tooth profile is represented as the *F* point; the *E* point is the tangent point of the tooth profile’s normal passing through the *F* point on the base circle [27].

In Figure 3, θ=∠FEG is the angle between the normal of point *F* and the positive direction of the *X*-axis. The equation for angle θ is:

(1) $$\theta \left(Z,\alpha ,\varphi \right)=\tan\left(\alpha \right)-inv\left(\alpha_{0}\right)+\varphi$$

The definitions of the parameters in Equation (1) are as mentioned earlier.

The roll length at pitch point C in Figure 3 is represented by $\rho_{0}$, then

(2) $$\rho_{0}=\left|NC\right|=\tan\left(\alpha_{0}\right)\cdot r_{b}$$

where $r_{b}$ is the radius of the base circle.

The equation for solving the roll length of any point on the profile is:

(3) $$\rho \left(\alpha \right)=\rho_{0}+\left(\tan\left(\alpha \right)-\tan\left(\alpha_{0}\right)\right)\cdot r_{b}$$

In the equation, $\rho \left(\alpha \right)$ represents the roll length at the point where the pressure angle on the profile is *α*.

A three-dimensional model of the double-flank process, when the angle of rotation is *φ*, can be generated using the tooth flank equations {X*_F_*, Y*_F_*, Z*_F_*} and the coordinate transformation matrix *M*_21_ [27].

(4) $$\left\{\begin{array}{l} X_{F}\left(Z,\alpha ,\varphi \right)=-r_{b}\cdot \sin\left(\theta \left(Z,\alpha ,\varphi \right)\right)+\rho \left(\alpha \right)\cdot \cos\left(\theta \left(Z,\alpha ,\varphi \right)\right)\\ Y_{F}\left(Z,\alpha ,\varphi \right)=r_{b}\cdot \cos\left(\theta \left(Z,\alpha ,\varphi \right)\right)+\rho \left(\alpha \right)\cdot \sin\left(\theta \left(Z,\alpha ,\varphi \right)\right)\\ Z_{F}\left(Z,\alpha ,\varphi \right)=Z \end{array}\right.$$

(5) $$M_{21}=\left[\begin{array}{cccc} -\cos\left(\varphi_{1}+\varphi_{2}\right) & -\cos\left(\pi /2+\varphi_{1}+\varphi_{2}\right) & 0 & a_{c}\cdot \cos\left(\varphi_{2}\right)\\ -\cos\left(\pi /2-\varphi_{1}-\varphi_{2}\right) & -\cos\left(\varphi_{1}+\varphi_{2}\right) & 0 & a_{c}\cdot \cos\left(\pi /2-\varphi_{2}\right)\\ 0 & 0 & 1 & 0\\ 0 & 0 & 0 & 1 \end{array}\right]$$

In Equation (5), $a_{c}$ is the linear encoder reading on the device shown in Figure 1, which is the center distance between the product gear and the master gear. $\varphi_{2}$ and $\varphi_{1}$ are the readings of two angular encoders, which are the rotation angles of the measured gear and the master gear.

By using the definition of the coordinate system, relevant formulas, and the simulation process in Figure 2 above, the simulation results can be calculated.

### 2.2. Experiment

In order to study the method of obtaining analytical parameters based on double-flank measurement, it is necessary to use the simulation method to obtain double-flank measurements of product gears with errors and master gears. The degree and form of the error in the product gear will have an impact on the double-flank measurements. By superimposing different types and amplitudes of profile deviations on the product gear for simulation tests, and observing whether the simulation results and the expected results are consistent, the correctness of the double-flank measurement simulation acquisition method proposed in this paper can be verified.

**Figure 4:**
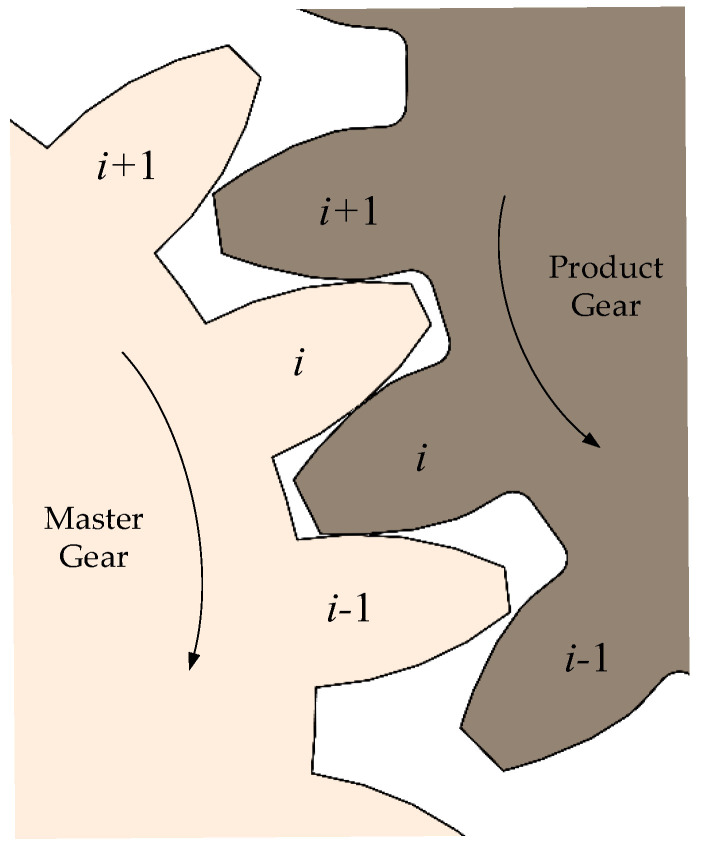
Schematic diagram of meshing of product gear with master gear [22].

The double-flank measurement process consists of the double-flank meshing of an individual tooth of the product gear. Taking the most common case where the contact ratio does not exceed 2 as an example, it is necessary to consider the joint influence of the meshing state of the current tooth, the previous tooth, and the subsequent tooth. Figure 4 shows the state of the product gear meshing with the master gear at the *i* − 1th, *i*th, and *i* + 1th teeth. The master gear is the driving gear and makes clockwise rotation. The product gear is the driven gear and makes counterclockwise rotation. This paper takes the meshing process of the master gear and product gear from the *i* − 1th tooth to the *i* + 1th tooth as an example to analyze the double-flank measurement process. Table 1 shows the basic parameters of the product gear and the master gear.

**Figure 5:**
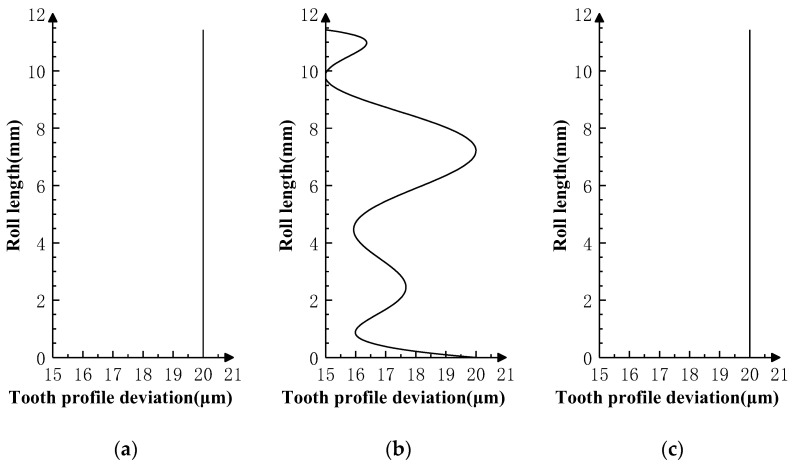
Profile deviations applied on three teeth in Simulation Experiment I. (**a**) Error of *i* − 1 tooth. (**b**) Error of *i* tooth. (**c**) Error of *i* + 1 tooth.

**Figure 6:**
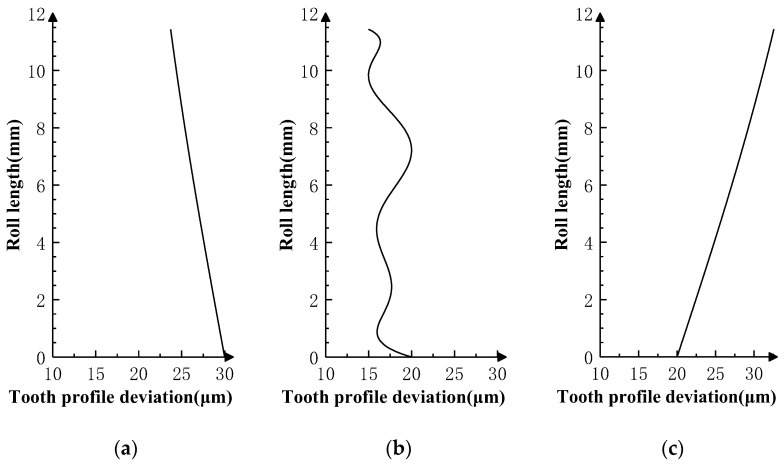
Profile deviations applied on three teeth in Simulation Experiment II. (**a**) Error of *i* − 1 tooth. (**b**) Error of *i* tooth. (**c**) Error of *i* + 1 tooth.

**Figure 7:**
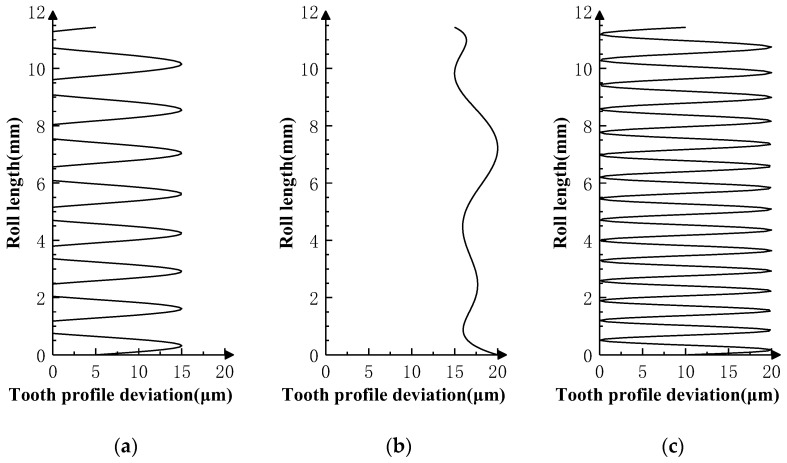
Profile deviations applied on three teeth in Simulation Experiment III. (**a**) Error of *i* − 1 tooth. (**b**) Error of *i* tooth. (**c**) Error of *i* + 1 tooth.

Superimpose the profile deviations shown in Figure 5, Figure 6 and Figure 7 on the *i* − 1th, *i*th, and *i* + 1th teeth of the product gear, respectively, and conduct three sets of double-flank simulation experiments according to the double-flank simulation method introduced earlier. The double-flank simulation results obtained take the form of time series of curves for the rotation angle and center distance of the two gears.

In the case of a product gear with profile deviations, the profile deviations will cause position deviation of the master gear from the theoretical position during double-flank meshing, which is manifested in the deviation of the rotation angle and X-direction displacement (i.e., center distance) of master gear. Therefore, the master gear rotation angle error and center distance variation in double-flank measurements contain information about profile deviations of product gear.

**Figure 8:**
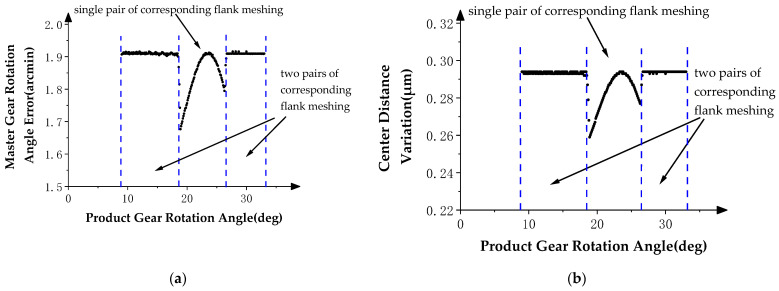
Double-flank measurements in Simulation Experiment I. (**a**) Master gear rotation angle error. (**b**) Center distance variation.

**Figure 9:**
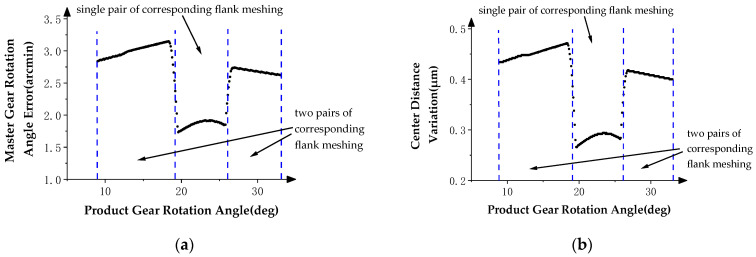
Double-flank measurements in Simulation Experiment II. (**a**) Master gear rotation angle error. (**b**) Center distance variation.

**Figure 10:**
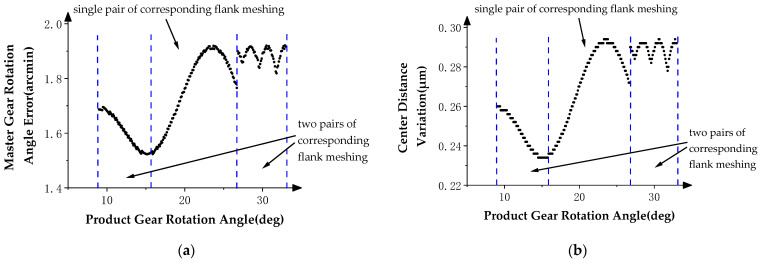
Double-flank measurements in Simulation Experiment III. (**a**) Master gear rotation angle error. (**b**) Center distance variation.

In order to study the influence of the profile deviations of the product gear in the double-flank process, the angle data measured by angle encoder 1 are subtracted from the theoretical rotation angle of the master gear to obtain the rotation angle error of the master gear. With these data, the final result of the double-flank measurement simulation is represented by the graph of the relationship between the above rotation angle error of the master gear, center distance variation of the gear pair, and the rotation angle of the product gear. In the curve, the rotation angle of the product gear is used as the horizontal axis, and the rotation angle error of the master gear and center distance variation are individually taken as the vertical axis direction, as shown in Figure 8, Figure 9 and Figure 10.

In order to better observe whether the simulation results meet the expectation that the profile deviations of the product gear have an impact on the double-flank process, in Figure 5, the theoretical profile deviations of the *i* − 1th tooth and *i* + 1th tooth are zero-order errors with an amplitude of 20 microns, and the theoretical profile deviation of the *i*th tooth is approximately the spline curve error of actual machining, with a maximum error amplitude of 20 microns.

Figure 8a shows the relationship between the rotation angle error of the master gear and the rotation angle of the product gear, and Figure 8b shows the relationship between the center distance variation of the gear pair and the rotation angle of the product gear. In Figure 8, the two curves show the same pattern: constant value errors in the two intervals at the beginning and end and fluctuating error values in the middle intervals of the measurement area. Compared with the profile deviation curves in Figure 5, it can be verified that the simulation results comply with the expectation that the measurements at the ends of the meshing process are influenced by profile deviations of neighboring teeth, while measurements in the middle part of the meshing process are mainly determined by profile deviations of the current tooth.

The theoretical profile deviations of the *i* − 1th and *i* + 1th teeth in Figure 6 are first-order errors with different inclination directions and amplitudes. The theoretical profile deviation of the *i*th tooth is a spline curve error that approximates actual machining, having a maximum error amplitude of 20 microns.

Figure 9a shows the relationship between the rotation angle error of the master gear and the rotation angle of the product gear, and Figure 9b shows the relationship between the center distance variation of the gear pair and the rotation angle of the product gear. In Figure 8, the two curves show the same pattern; in the two intervals at the beginning and end, there are first-order error values similar to the theoretical profile deviations of the *i* − 1th tooth and the *i* + 1th tooth in inclination direction and amplitude, and fluctuating error values appear in the middle interval of the measurement area. By comparing the profile deviations curve in Figure 6, it can be verified that the simulation results meet the same experimental expectations as Simulation Experiment I.

The theoretical profile deviations of the *i* − 1th and *i* + 1th teeth in Figure 7 are high-order sine errors with different frequencies and amplitudes. The theoretical profile deviation of the *i*th tooth is a spline curve error that approximates actual machining, with a maximum error amplitude of 20 microns.

Figure 10a shows the relationship between the rotation angle error of the master gear and the rotation angle of the product gear, and Figure 10b shows the relationship between the center distance variation of the gear pair and the rotation angle of the product gear. In Figure 10, the two curves show the same pattern; in the two intervals at the beginning and end, there are high-order sine error values similar to the theoretical profile deviations of the *i* − 1th tooth and *i* + 1th tooth in frequencies and amplitude. There are no high-frequency errors at the middle and left end of the measurement area, except error values that conform to the spline curve error. By comparing the profile deviation curve in Figure 7, it can be verified that the simulation results meet the experimental expectation, that is, profile deviations of the *i* − 1th tooth do not have an impact on the position deviation of the master gear during the double-flank process of the experiment due to the fact that the amplitude of the high-order error of the *i* − 1th tooth is generally smaller than the spline curve error of the *i*th tooth.

## 3. Solving Method of Analytical Parameters

### 3.1. Method

**Figure 11:**
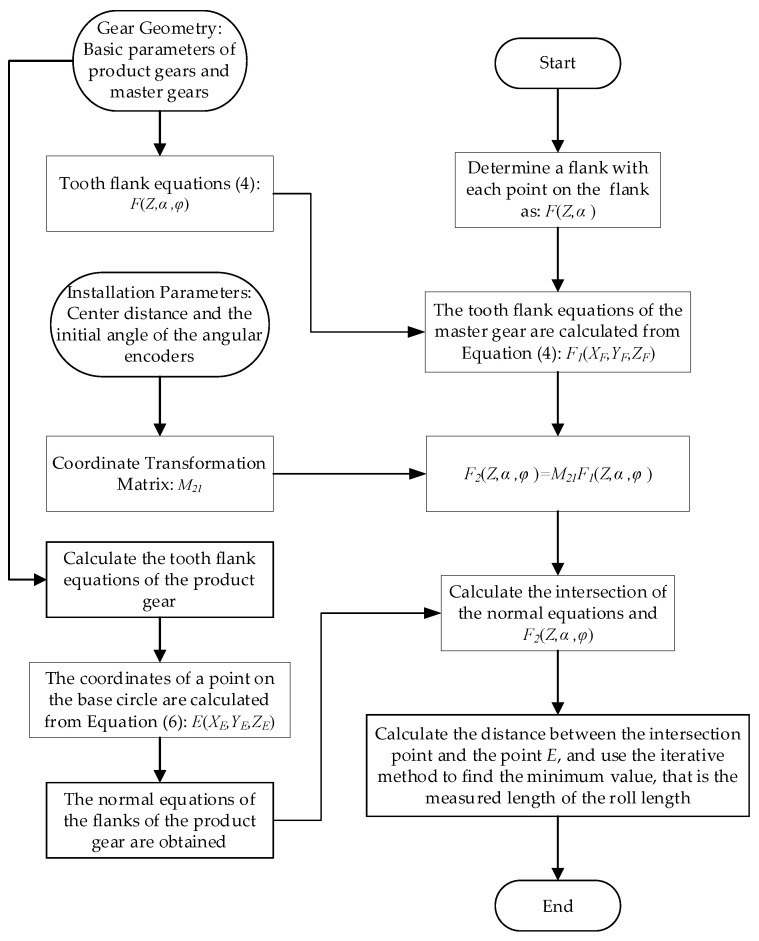
Flow chart of solving the actual profile of the product gear.

Based on the double-flank measurements above, this paper proposes methods to solve analytical parameters such as profile deviations and pitch deviations, with the flow chart of the calculation shown in Figure 11. Firstly, according to the known sensor readings of the double-flank measurements, transform the master gear flank equation to the coordinate system of the product gear through the coordinate transformation matrix (Equation (5)). Then, calculate the coordinates of the intersection point between the normal *EF* and the flank of the master gear. Calculate the distance between the intersection point and point *E*, and use iterative methods to obtain the roll length, then obtain the actual profile of the product gear. Finally, by analyzing the roll length error, the analytical parameters of the product gear can be obtained. 

The coordinate equation of the tangent point *E* (X*_E_*, Y*_E_*, Z*_E_*) between the normal of any point *F* on the profile and the base circle is [27]:

(6) $$\left\{\begin{array}{l} X_{E}\left(Z,\alpha ,\varphi \right)=-r_{b}\cdot \sin\left(\theta \left(Z,\alpha ,\varphi \right)\right)\\ Y_{E}\left(Z,\alpha ,\varphi \right)=r_{b}\cdot \cos\left(\theta \left(Z,\alpha ,\varphi \right)\right)\\ Z_{E}\left(Z,\alpha ,\varphi \right)=Z \end{array}\right.$$

With the coordinate equation of point *E* on the base circle in Equation (6) and the coordinate equation of any point *F* (X*_F_*, Y*_F_*, Z*_F_*) on the profile in Equation (4), the following unique profile normal equation corresponding to point *F* can be obtained:

(7) $$\left\{\begin{array}{l} Y-Y_{F}=X\cdot \frac{\left(Y_{F}-Y_{E}\right)}{\left(X_{F}-X_{E}\right)}-X_{F}\\ Z=Z_{F} \end{array}\right.$$

According to Equation (7), point *F* on a theoretical profile of the product gear can determine a unique normal. The intersection point between this normal and the paired profile on the master gear during meshing is *S*. At a certain moment, after substituting the parameter equation (Equation (4)) of the points on the profile of the master gear into Equation (7), and limiting the range of the pressure angle to the range of the base circle pressure angle to the tip circle pressure angle, a unique solution can be obtained. This solution is the coordinates of the intersection point between the normal and the paired profile on the master gear at this moment in the gear meshing process. Corresponding to different moments, multiple intersections *S* (t) of the normal with the profile of the master gear can be obtained, and the distance between these intersections *S* (t) and the point *E* is the maximum possible roll length of the point *F* at each moment, written as

(8) $$\rho_{\max}(t)=\left|ES(t)\right|$$

Use the iterative method to select the minimum value of the maximum possible extension at all times during the gear meshing process, written as

(9) $$\rho_{\min\max}=\min(\rho_{\max}(t))$$

The $\rho_{\min\max}$ is the actual roll length measured at point *F*.

With the above method, the actual roll length of any point on the profile of the product gear can be calculated. Additionally, according to the obtained roll length of each point of the profile, the profile calculation results of the product gear can be obtained. 

During the double-flank process, the contact ratio of the corresponding flank may be greater than 1, which means that there is a state switch between the single pair of flank contacts and two pairs of flank contacts on the corresponding flank. Therefore, the profile calculation results of the product gear based on the double-flank measurements are correct only in partial areas, that is, the area where the number of teeth on the corresponding flank participating in meshing is equal to 1. This judgment has been proven in Experiments I, II, and III.

### 3.2. Experiments

**Figure 12:**
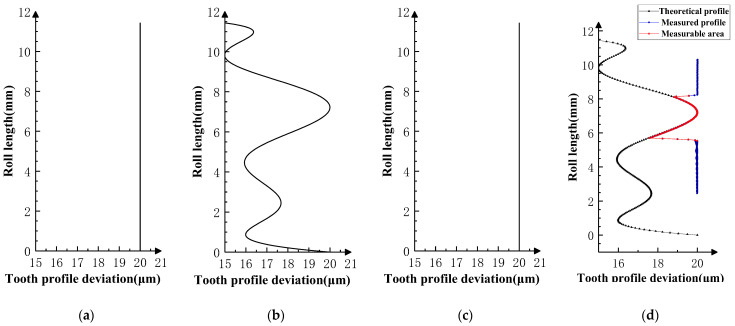
Experiment I (front and rear teeth have zero-order error) [22]. (**a**) Error of *i* − 1th tooth. (**b**) Error of *i*th tooth. (**c**) Error of *i* + 1th tooth. (**d**) Profile measurements and measurable area.

**Figure 13:**
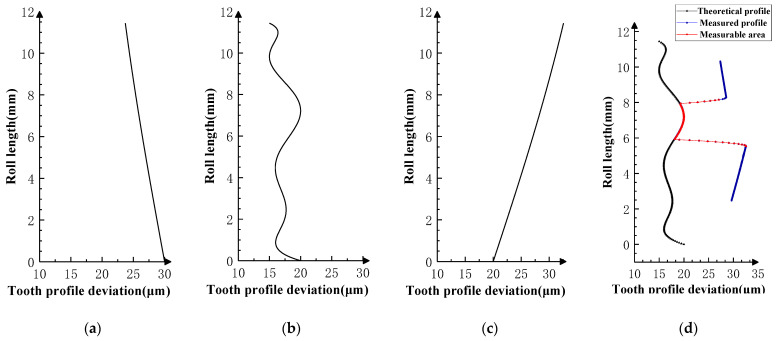
Experiment II (front and rear teeth have first-order error). (**a**) Error of *i* − 1th tooth. (**b**) Error of *i*th tooth. (**c**) Error of *i* + 1th tooth. (**d**) Profile measurements and measurable area.

**Figure 14:**
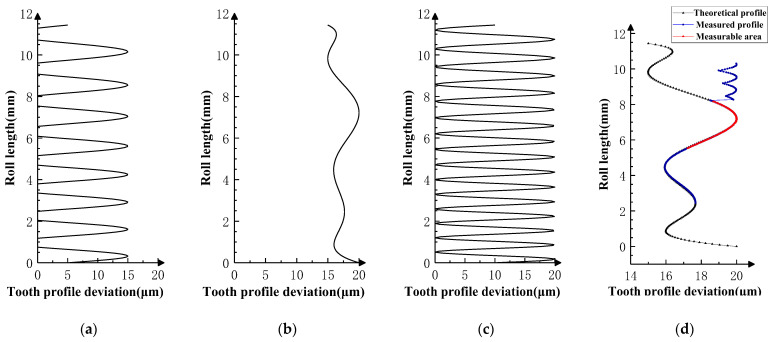
Experiment III (front and rear teeth have high-order error). (**a**) Error of *i* − 1th tooth. (**b**) Error of *i*th tooth. (**c**) Error of *i* + 1th tooth. (**d**) Profile measurements and measurable area.

Based on the double-flank measurements of the three sets of simulation experiments in Figure 8 and Figure 10, the profile deviations in Figure 12, Figure 13 and Figure 14 can be obtained by using the above solving method for analytical parameters. Among them, Figure 12 corresponds to Experiment I, where the profile deviations of the front and rear teeth are zero-order errors; Figure 13 corresponds to Experiment II, where the profile deviations of the front and rear teeth are first-order errors; and Figure 14 corresponds to Experiment III, where the profile deviations of the front and rear teeth are high-order errors.

The measurable region marked In the measurements of Experiments I, II, and III Is the single-flank meshing region of the corresponding profile of the product gear in the double-flank measurement [22]. This region is calculated by geometrical solving methods based on the rule of contact ratio in the double-flank process. The calculated results of the product gear profile in the measurable region should be reliable. However, in the unmeasurable area, the product gear has more than one flank participating in meshing at the same time. Therefore, the calculated profile of the current tooth may differ from the actual profile.

The results of the measurement in Experiments I, II, and III are categorized into theoretical profile deviations and measured profile deviations. The relationship between the measured profile deviations in the measurable area and the theoretical profile deviations of the target tooth (*i*th tooth) is identical (the difference in profile deviations between the two does not exceed 0.03 μm), which proves that the measured profile deviations in the measurable area truly reflect the profile deviations of the measured tooth. The two ends of the measurable region show different characteristics based on the frequency and amplitude of the theoretical profile deviation of the front and back neighboring teeth. It proves that the profile deviation measured outside the measurable region cannot truly reflect the profile deviations of the measured teeth, and that it belongs to the unmeasurable area of the analytical parameters in the process of double-flank measurement. 

After obtaining the measured data of the profile in the measurable region, the pitch deviations of the product gear can be calculated according to the relevant gear accuracy standards, including the single pitch deviations fp, the total tooth pitch deviations Fp, etc. [28]. Overall, the method presented in this paper can be used to obtain analytical gear accuracy parameters based on the double-flank measurement data.

## 4. Conclusions

A simulation method for double-flank measurement of a gear with errors and a solving method for obtaining analytical parameters are proposed in this paper. In the double-flank simulation method, the double-flank measurements of a gear with errors can be obtained by using stepwise approximation and collision detection algorithms. In the solving method, based on double-flank measurements, the minimum rolling length is calculated by iteration to obtain the analytical parameters of gear accuracy. Therefore, using these methods can enhance the application value of double-flank measurements. 

The correctness of these two methods is verified by several experiments.

(1) The experiment data of simulation method show that reasonable double-flank measurements can be obtained by the double-flank simulation method proposed in this paper when there are zero-order, first-order, high-order, and spline-curve-shaped deviations in the profile of the product gear. It is verified in the experiment that the measurements at the ends of the meshing process are influenced by profile deviations of neighboring teeth, while measurements in the middle part of the meshing process are mainly determined by profile deviations of the current tooth, which complies with the expectation;
(2) The experiment data of solving method show that part of the profile deviations of the measured gear can be obtained. The measured profile deviations in the measurable area truly reflect the profile deviations of the measured tooth. The two ends of the measurable region show different characteristics based on the frequency and amplitude of the theoretical profile deviation of the front and back neighboring teeth.

In the methods and experiments of this paper, the spur gear is taken as the research object. Using the methods in this paper, or by slightly improving them, the analytical parameters of a spiral gear and other types of gears can also be calculated. In the future, relevant experiments will be conducted under actual measurement conditions to further verify the correctness and application value of the method proposed in this paper.

## Figures and Tables

**Table 1:** Gear parameters used in the experiment [22].

Parameter	Modulus (mm)	Number of Teeth	Transverse Pressure Angle (deg)	Profile Shift Coefficient
Product gear	2	20	20	0
Master gear	2	19	20	0

## Data Availability

The data that support the findings of this study are available from the corresponding author upon reasonable request.
